# Determinants of self-rated health among Venezuelan migrant women in Brazil: a cross-sectional study

**DOI:** 10.1016/j.lana.2025.101077

**Published:** 2025-04-15

**Authors:** Maria do Carmo Leal, Thaiza Dutra Gomes de Carvalho, Yammê Ramos Portella Santos, Rita Suely Bacuri de Queiroz, Paula Andrea Morelli Fonseca, Antônio Augusto Moura da Silva, Celia Landmann Szwarcwald, Pía Riggirozzi

**Affiliations:** a National School of Public Health, Oswaldo Cruz Foundation, Rio de Janeiro, RJ, Brazil; b Leônidas and Maria Deane Institute, Oswaldo Cruz Foundation, Manaus, AM, Brazil; c Center for Biological and Health Sciences, Department of Public Health, Federal University of Maranhão, São Luís, MA, Brazil; d Institute of Scientific and Technological Communication and Information in Health, Oswaldo Cruz Foundation, Rio de Janeiro, RJ, Brazil; e Department of Politics and International Relations, University of Southampton, Highfield Campus, Southampton, UK

**Keywords:** Brazil, Venezuela, Women’s health, Self-rated health, Transnational motherhood

## Abstract

**Background:**

Migration between countries in the Global South remains under-researched, with even less focus on the intersections of migration, transnational motherhood, and health. This study examines factors that impact health self-perception among Venezuelan migrant women in Brazil.

**Methods:**

A cross-sectional study was conducted using Respondent-Driven Sampling with 2012 Venezuelan migrant women, aged 15 to 49, who migrated to Brazil between 2018 and 2021. A hierarchical logistic regression model was applied, calculating crude and adjusted odds ratios with 95% confidence intervals.

**Findings:**

About a third of migrant women left children in Venezuela and experienced violence during migration. 73% were aged 15–34, 70% had completed high school, 66% identified as mixed-race, and 75% lacked paid work in the previous month. Nearly a quarter received government financial aid; most had been in Brazil for a year or less. While they rated their health better in Brazil than in Venezuela, multivariate analysis shows poorer self-rated health is strongly linked to leaving children behind and experiencing violence.

**Interpretation:**

Venezuelan migrant women report better self-assessed health upon arrival in Brazil, likely due to improved access to essential services and safety. However, this perception declines due to stressors such as separation from children, economic hardship, and violence during migration and in Brazil, with poorer self-rated health linked to transnational motherhood and violence. This underscores the cumulative impact of these challenges and the need for targeted policies to address them.

**Funding:**

This work was supported by the UK 10.13039/501100000269Economic and Social Research Council (ES/T00441X/1).

**Research in context**

**Evidence before this study**

A literature review was conducted based on PubMed/Medline, SCIELO and SCOPUS combining the following terms to create the search strategy ((Latin America) AND (human migration)) AND ((((Health Social Determinants of Health) OR Women’s Health) OR Health Status) OR Mental Health) using Mesh terms and other fields. Searching for studies on the self-rated health of migrant women in the global South up to April 2024. This literature review was complemented by a review of books on the topic and analysis of references in identified articles and knowledge of the authors themselves. We found around 300 studies, but only a few of them included self-rated health evaluations of migrant women, including a scoping review that focused on mental health and these studies were carried out in developed host countries. None of them focus on transnational motherhood and violence on the mother’s self-assessment health. Although we find few studies focus on the children’s or parent’s health that was left behind for mothers and it was not in South-South migration. We concluded that there are no published studies about Venezuelan migration to Brazil that include subjective health assessments from migrant women that provide insights into gendered dimensions of the health of migrants beyond medical considerations and measures. While self-rated health is frequently used in epidemiological and public health research as a valuable tool for assessing and predicting health outcomes in populations, migrant women’s self-perception of health and its interconnections with displacement-related experiences, especially related to gender violence, irregular migration during the COVID-19 pandemic, and transnational families are still unexplored. Forced migrations between countries of the global South, especially in South America, are little studied, most of them in specific groups, without population representativeness.

**Added value of this study**

This work uses a sampling technique specifically designed for hard-to-reach migrant populations. The study is representative, involving interviews with 2012 Venezuelan migrant women in two northern Brazilian capitals, which host the largest number of Venezuelan migrants in the country. It identifies and examines gender-related factors in the migration process, such as transnational motherhood and experiences of violence, and how these factors influence subjective health assessments. Since self-rated health is a powerful and consistent predictor of future health events, including mortality, the insights gained from this study are particularly significant.

**Implications of all the available evidence**

The article provides evidence on the suffering of migrant women with gender violence, experienced in the migratory path and in Brazil, which interfered with their self-perception of health. It also showed an association of poorer self-rated health with the experience of transnational motherhood and draws attention to the urgency for human rights policies that fight violence against women, in Latin America and the promotion of family reunification of migrants in the receiving country.

## Introduction

Venezuela has experienced an enduring, systemic, and multidimensional crisis that led to a massive displacement of 7.7 million people since 2015. More than 6.5 million reside in Latin America, being Brazil the third host country after Colombia and Peru.^1^ Due to escalating threats and physical insecurity, including health-related issues, many women have found that leaving their home country is the only option to ensure their safety and that of their families. Furthermore, many are forced to make decisions and act for the well-being of their loved ones, including having to choose which children they take with them in the migratory journey and which ones are left behind. They continue to bear care responsibilities for those left behind through transnational means, such as sending remittances or finding other precarious ways to save money for a future family reunion.

There is increasing attention on the gendered dimensions of displacement, highlighting how displacement heightens vulnerabilities and exposes migrants, especially forced migrants, to greater risks and ill-health. Women and girls face compounding risks during displacement, particularly sexual abuse, violence, trafficking and labour exploitation.^2^^,^^3^ These risks persist in their new places of residence due to barriers related to poverty, stigma, discrimination, social exclusion, language and cultural differences, socio-cultural norms, and legal status, all of which act as health determinants for migrants.^4^, ^5^, ^6^ However, less attention has been placed on how the migratory process impacts migrant women’s self-perception of health.

Self-perception of health status is a subjective indicator, a multidimensional construct that consists of individuals’ perception of their health, involving physical and emotional components, as well as social, and well-being aspects that may affect the satisfaction with their own life.^7^^,^^8^ Usually measured by a single question, it is widely used by epidemiology because it is reliable for assessing health status and useful for studies of health inequities among different populations. While it may not capture all aspects of a person’s health, the indicators have commonly been used as a predictor of various health outcomes in several studies that demonstrate that individuals who report poor self-rated health are more likely to experience adverse health outcomes, including higher rates of morbidity, hospitalisations, and mortality.^9^, ^10^, ^11^ The self-perception of health indicators among migrants can vary based on various factors, including individual experiences, cultural background, socioeconomic status, and access to healthcare.

Understanding how the migration process can impact migrants’ self-perception of health becomes essential to provide evidence that can help propose and support specific public health policies for this population.

This study aimed to describe the self-assessed health of Venezuelan women who migrated to Brazil between 2018 and 2021 and explore factors associated with worse levels of self-perceived health among them.

## Methods

This cross-sectional study utilised primary data collected through a survey carried out within the scope of an ESRC-funded project, titled *Redressing Gendered Health Inequalities of Displaced Women and Girls in Contexts of Protracted Crisis in Central and South America (REGHID).* Data collection took place between July and September 2021. We interviewed 2012 Venezuelan migrants in Brazil, 755 women in Manaus and 1257 in Boa Vista. These two capitals of the Amazonas and Roraima states respectively, were chosen because they are the largest and closest cities to the border, receiving the highest number of Venezuelans in the country. The minimum sample size was estimated at 730 valid interviews per city. This size was calculated to estimate the percentage of migrants who received health-related care in the 15 days prior to the survey. We used the proportion of 19% from the National Health Survey in Brazil, carried out in 2019^12^ and a bilateral error of 4%, 95% confidence. Also, a design effect of 2 was considered in the sample size estimation.^13^

The Respondent Driven Sampling (RDS) method was used to establish the sample. Six seeds were selected in Manaus and nine in Boa Vista, and started with women considered leaders of different social groups among Venezuelans. They received the first three invitations and were responsible for passing them on to three women in their network. Each of these women received three more invitations that were passed on with the same rule, and so on until the sample size of the study was reached in each city. The eligibility criteria to participate in the research were: a) being a national from Venezuela; b) self-identified as a woman; c) having arrived in Brazil within three years (between 2018 and 2021); d) being invited to take part in the survey by the research team; e) being the first time to participate in the research.

All interviewed women were asked about the size of their social networks. This information was used to calculate the probability of each participant being selected for the study. Each network was initiated by a “seed” participant. Each cluster was made up of the recruits of each recruiter, and each city was treated as a stratum. All interviews were conducted in person with support from local NGOs in each city, *Hermanitos* in Manaus and *Mexendo a Panela* in Boa Vista. These organisations facilitated access to Venezuelan migrant women and provided private, safe interview spaces. The interviews were conducted by Venezuelan women who had been trained by the Fiocruz team to administer the questionnaire. This questionnaire, designed specifically for this study, consisted solely of closed-ended questions.

For the analyses presented in this article, self-rated health was considered as the outcome. This variable was measured by the following question: “Considering health as a state of physical and mental well-being, and not just the absence of disease, how to assess your health status?” 1, Very good | 2, Good | 3, Regular | 4, Bad | 5, Very Bad | 99, Don’t know/Don’t answer. A ‘Good self-rated health’ included the response categories Very Good and Good, and ‘Poor self-rated health’ the categories: Fair, Poor and Very Poor.

We also developed a structured theoretical model using an adaptation of the classification proposed by Fuchs et al. (1996).^14^ In this model, the explanatory variables were arranged by the authors at hierarchy levels according to their relationship with the outcome (distal, intermediate, and proximal). The explanatory variables were classified into structural or contextual factors; psychosocial and behavioural factors; and individual factors.^15^

Distal level–The following explanatory variables were selected to describe the contextual factors: education level, racial/ethnic identity, household and family income in the last month, paid work in the last month; marital status; type of residence, time living in Brazil and migratory status. To measure racial/ethnic identity, we used the self-reported classification used in the Brazilian census, carried out by the Brazilian Institute of Geography and Statistics—IBGE (IBGE).^16^

Intermediate level—Psychosocial and behavioural factors were included at this level. To represent the psychosocial factors, the following variables were used: experience of an episode of violence; having to leave a child(ren) in Venezuela; migrating alone to Brazil; and having to pay for facilities to enter Brazil. To represent behavioural factors, a healthy lifestyle variable was created, which included women who reported not consuming tobacco and/or alcohol.

Proximal level—At this level, individual and/or biological characteristics were included. To represent this level, the following variables were used: age, parity, current pregnancy, and disease diagnosis.

Absolute and relative frequencies were calculated according to the assessment of health status. In the bivariate analyses, Pearson’s chi-square test was used to assess the association between the explanatory variables tested and the self-rated health outcome. All variables that presented a p-value ≤ 0.20 in the bivariate analyses were included in the construction of a hierarchy logistic regression model.

At each level, the variables were also selected with a criterion of p < 0.20 to remain in the model. For the following analyses (with the inclusion of variables from a new level), the variables that presented an association (p < 0.05) with poor self-rated health were kept in the model after adjusting for the variables of the same level and of the higher hierarchical levels. Crude and adjusted odds ratios and 95% confidence intervals (95% CI) were calculated.

### Sensitivity analysis

In RDS, the tendency of a participant to invite people with similar characteristics is known as homophily.^17^ To assess whether this effect was influencing the self-rated health outcome studied, a logistic regression model was used with the self-assessment of the recruiters as proposed by Landmann^18^ and the effect of homophily was not observed in the data.

All analyses considered complex sampling. The software used was SPSS, version 23.

### Ethics considerations

This study involves human participants and was carried out in accordance with the relevant guidelines and regulations of the Declaration of Helsinki and was approved by Research Ethics Committee of the Federal University of Maranhão (Certificate of Submission for Ethical Evaluation number 35617020.9.1001.5087). All participants received and signed a written informed consent form before the interview.

### Role of the funding source

The funder (Economic and Social Research Council- ESRC UK) had no involvement in the study design; collection, analysis and interpretation of data; writing of the paper; or in the decision to submit the paper for publication.

## Results

A total of 2012 Venezuelan women were recruited for this study—755 in Manaus and 1257 in Boa Vista—capturing a variety of demographic and migratory characteristics among Venezuelan migrant women who entered Brazil between 2018 and 2021. The complete sample (N = 2012) was included in the final analysis.

Nearly two-thirds of the interviewed women were under 34 years old, had completed high school (69.9%–1406/2012), self-identified as mixed-race (66.1%–1322/2012), and had a partner (61.3%- 1233/2012). The majority had not held a paid job in the previous month (75.7%–1523/2012) and had arrived in Brazil within the past year (66.2%–1333/2012), while 21.4% (429/2012) received some form of government financial aid. Most of the migrant women (62.0%, 1247/2012) lived in collective housing, and only 10.2% (199/1897) were in an irregular migratory status. Among them, 87.5% (1760/2012) migrated with companions, 38.5% (771/2012) paid for assistance to enter Brazil, and 13.3% (267/2012) experienced some form of violence during migration or after arrival. Additionally, 31.2% (536/1718) of the women interviewed had to leave one or more of their children behind in Venezuela (Table 1).

**Table 1:** Sociodemographic, health, and behavioural characteristics of Venezuelan women who migrated to Brazil between 2018 and 2021, by self-rated health.

	Good^a^		Poor^a^		Total		p-value^b^
	N	%	N	%	N	%	
**Contextual factors**							
Educational level							0.12
Elementary	221	69.6	98	30.4	317	15.8	
High School	1031	73.3	375	26.7	1406	69.9	
College	192	66.4	97	33.6	289	14.4	
Race/ethnicity							0.02
White	412	72.7	155	27.3	567	28.3	
Brown	933	70.6	389	29.4	1322	66.1	
Black	52	80.0	13	20.0	65	3.3	
Indígenous	43	91.6	4	8.4	47	2.3	
Marital status							0.03
No partner	586	75.2	194	24.8	779	38.7	
Have a partner	857	69.6	375	30.4	1233	61.3	
Paid work in the last month							0.12
Yes	343	70.2	146	29.8	489	24.3.	
Receives government financial assistance							0.05
Yes	293	68.3	136	31.7	429	21.4	
Housing type							0.74
Collective housing	898	72.0	349	28.0	1247	62.0	
Familiar housing	543	71.2	220	28.8	763	38.0	
Time of arrival in Brazil							0.07
Up to 1 year	975	73.1	358	26.9	1333	66.2	
Over 1 year	468	68.9	211	31.1	679	33.8	
Migratory status							0.71
Asylum seeker	601	71.5	239	28.5	840	42.9	
Resident^c^	632	73.7	226	26.3	858	43.9	
Irregular	187	72.1	12	27.9	199	10.2	
**Psychosocial factors**							
Migrated alone							0.56
Yes	185	73.5	67	26.5	252	12.5	
Paid for facilities to enter Brazil							0.05
Yes	528	68.5	243	31.5.	771	38.3	
Episode of violence on the route or in Brazil							0.00
Yes	156	58.3	111	41.7.	267	13.3	
Left child in Venezuela							0.00
Yes	343	64.0.	193	36.0.	536	31.2	
**Behavioural factors**							
Lifestyle							0.05
Health	1259	72.7	472	27.3	1731	86.0	
Not healthy	184	65.6	97	34.4	281	14.0	
**Individual/biological factors**							
Age							0.00
15–24 years	576	76.2	180	23.8	756	37.6	
25–34 years	515	71.5	205	28.5	721	35.8	
35–49 years	309	65.7	152	34.3	484	24.1	
Parity							0.12
None	397	73.5	143	26.5	540	26.8	
1 or 2 children	562	73.7	201	26.3	763	37.9	
3 or more children	484	68.2	225	31.8	709	35.2	
Actual pregnancy							0.39
Yes	83	67.6	40	32.4	123	6.1	
Disease diagnostic							0.00
Yes	252	55.1	206	44.9	458	22.8	

OR: Odds Ratio, CI Confidence Interval.

a ‘Good self-rated health’ included categories Very Good and Good, and ‘Poor self-rated health’: Fair, Poor and Very Poor.

b Chi-square test: evaluates the association between self-rated health and sociodemographic, health, and behavioural characteristics.

c Resident included temporary and permanent.

In the self-assessment survey, Venezuelan women reported a significantly higher percentage of good self-rated health after they arrived in Brazil (71.7%; [95% CI: 69.3%, 74.0%]) compared to when they were living in Venezuela (58.1%; [95% CI: 55.5%, 60.6%]).

### Contextual factors: distal level

At this level, socioeconomic factors and variables related to the migration situation in Brazil were included: migratory status and time in the country. The status is directly related to access to essential services, such as the labour market and health. Time is important for adapting to local society.

All included variables met the criterion of association (p-value < 0.20) with the outcome of poor self-rated health when evaluated in isolation, except for paid work, housing type, and migratory status. After adjusting for the model’s distal variables, only educational level, race/ethnicity, and marital status remained statistically significant (p < 0.005) and were included in the adjustments for the next levels. Among those with a partner, there was a higher likelihood of poor self-assessment (OR 1.33 [95% CI: 1.04, 1.72]), whereas those who identified as indigenous had a lower likelihood of poor self-assessment (OR 0.25 [95% CI: 0.09, 0.66]) (Table 2).

**Table 2:** Hierarchy model of factors associated with poor self-rated health among Venezuelan women who migrated to Brazil between 2018 and 2021, distal level.

	Distal level	OR crude	CI (95%) OR crude		OR adjusted	CI (95%) OR adjusted distal level	
**Contextual factors**	**Educational level**						
	Without college	1	_	_	1	_	_
	College	1.34	0.97	1.85	1.308	0.936	1.828
	**Race/ethnicity**						
	White	1	_	_		_	_
	Brown	1.11	0.85	1.45	1.10	0.84	1.45
	Black	0.67	0.34	1.32	0.71	0.35	1.43
	Indígenous	0.24	0.09	0.64	0.25	0.09	0.66
	**Marital status**						
	No partner	1	_	_	1.00		
	Have a partner	1.32	1.03	1.70	1.33	1.04	1.72
	**Paid work in the last month**						
	No	0.91	0.70	1.18	_	_	_
	**Receives government financial assistance**						
	Yes	1.23	0.94	1.61	1.09	0.78	1.50
	**Housing type**						
	Collective housing	0.96	0.76	1.22	_	_	_
	Familiar housing	1	_	_	_	_	_
	**Time of arrival in Brazil**						
	up to 1 year	0.82	0.64	1.04	0.887	0.66	1.18
	over 1 year	1			1		
	**Migratory Status**						
	Asylum seeker	1.03	0.70	1.51	_	_	_
	Resident^a^	0.92	0.63	1.35	_	_	_
	Irregular	1	_	_	_	_	_

OR: Odds Ratio, CI Confidence Interval.

a Resident included temporary and permanent.

### Psychosocial and behavioural factors—intermediate level

The psychosocial factors tested included issues related to migratory vulnerability, which has a direct impact on women’s mental health, and behavioural lifestyle factors (smoking and alcohol use). All variables tested were associated with poor self-rated health in the unadjusted model, except for having migrated alone (p < 0.20). When including in the model all the confounding factors of the intermediate level (column 2) and the intermediate and distal level (column 3), the lifestyle variable became non-significant (p > 0.05). There is a higher chance of negative self-rated health among those who paid to enter in Brazil (OR 1.43 [95% CI: 1.09, 0.88]; those who reported having suffered some type of violence during their migratory journey or after arrival in the country (OR 1.97 [95% CI: 1.41, 2.76], and those who had to leave children in Venezuela (OR 1.36 [95% CI: 1.02, 1.81]), referred to in the literature as transnational mothers (Table 3).

**Table 3:** Hierarchy model of factors associated with poor self-rated health among Venezuelan women who migrated to Brazil between 2018 and 2021, intermediate level.

	Intermediate level	OR crude	CI (95%) OR crude		OR adjusted for intermediate factors	CI (95%) OR for intermediate factors		OR adjusted for intermediate and distal factors	CI (95%) OR adjusted for intermediate and distal factors	
**Contextual factors**	Migrated alone	0.90	0.64	1.27	_	_	_	_	_	_
	Paid for facilities to enter Brazil	1.29	1.00	1.65	1.40	1.07	1.84	1.43	1.09	1.88
	Episode of violence on the route or in Brazil	2.01	1.47	2.74	2.01	1.44	2.81	1.97	1.41	2.76
	Left child in Venezuela	1.40	1.05	1.85	1.36	1.02	1.81	1.36	1.02	1.81
**Behavioural factors**	Not healthy Lifestyle	1.40	1.01	1.94	1.24	0.88	1.76	1.23	0.87	1.75

OR: Odds Ratio, CI Confidence Interval.

### Individual/biological factors—proximal level

Proximal level factors included individual and biological characteristics and variables related to reproductive health: pregnancy at the moment of the interview and parity. All factors were associated with poor self-rated health in the unadjusted model (column 1), except for those who were pregnant at the time of the interview. When adjusting the model for the variables at the distal level (column 2), the age variable lost statistical significance. After adjusting for both distal and intermediate levels (column 3), the model identified the following variables as significantly associated with the outcome: disease diagnosis and parity, with an OR of 0.54 [95% CI: 0.36, 0.82] for two children and an OR of 0.61 [95% CI: 0.40, 0.95] for three or more children. Having children acts as a protective factor, reducing the chances of poor self-rated health when compared to childless migrant women (0.544 [95% CI: 0.36, 0.82]), and the diagnosis of illness increases the chances of poor self-rated (2.40 [95% CI: 1.79, 3.22]) (Table 4).

**Table 4:** Hierarchy model of factors associated with poor self-rated health among Venezuelan women who migrated to Brazil between 2018 and 2021, proximal level.

	Proximal Level	OR crude	CI (95%) OR crude		OR adjusted proximal level	CI (95%) OR adjusted proximal level		OR adjusted for distal, intermediate and proximal levels	CI (95%) OR adjusted for distal, intermediate and proximal levels	
**Individual/biological factors**	**Age**	1.03	1.01	1.04	1.02	1.01	1.04	1.01	0.99	1.03
	**Actual pregnancy**									
	Yes	1.23	0.77	1.96	_	_	_	_	_	_
	**Parity**									
	1 or 2 children	0.99	0.73	1.35	0.88	0.64	1.21	0.54	0.36	0.82
	3 or more children	1.29	0.95	1.75	0.97	0.68	1.38	0.61	0.40	0.95
	**Disease diagnostic**									
	Yes	2.68	2.05	3.50	2.54	1.94	3.327	2.40	1.79	3.22

OR: Odds Ratio, CI Confidence Interval.

## Discussion

This study aimed to investigate the factors influencing self-perception of health among Venezuelan migrant women in Brazil. The findings indicate that experiences of violence, both during migration and after arrival in Brazil, as well as disease diagnosis and the emotional and social stressors related to leaving children behind, are strongly associated with poorer self-rated health. In addition, sociodemographic factors such as education, race/ethnicity, marital status, and psychosocial stressors, including the challenges of migration and caregiving from afar, play significant roles in shaping health perceptions.

These findings are situated within the broader context of migration patterns and the reasons driving Venezuelan women to seek refuge in Brazil. According to a survey by Do Carmo Leal et al.,^14^ the main motivations for migration are the severe challenges of food insecurity (54%) and limited access to healthcare (37.8%) in Venezuela. These hardships, alongside the trauma and stress of migration, likely exacerbate the health issues faced by women, influencing how they perceive their well-being once they arrive in Brazil.

Many forced migrants flee dangerous conditions that affect their health and access to health care, and, in many ways, displaced populations are at risk and exposed to unique and complex health needs that require attention both upon arrival and throughout the settlement process. Brazil has a distinctive approach to migrants and refugees, who are entitled to the same rights and access to public services as nationals, including the right to health, education and the right to shelter as universally recognised entitlements in its Constitution.^5^^,^^19^ Therefore, in Brazil, all migrants are guaranteed access to the public, universal and free health service through the Universal Health System (SUS).^20^^,^^21^ This may explain in part the fact that Brazil, the only country in Latin America whose primary language is Portuguese, not Spanish, rose from fifth to third in the ranking of host countries to Venezuelan-displaced people in the last three years.^22^ This may also explain the increasing attention that this bilateral migration has prompted in the study of migration and health.^5^^,^^23^^,^^24^ What became apparent in the literature is the need to address sexual and reproductive health in humanitarian settings as well as the challenges and facilitators that affect access to health services and healthcare during migration and in settlement, particularly for forced migrants. Less apparent is how the decisions many women and girls are forced to make in response to and cope with the risks faced during displacement may themselves create new health risks, and how those are perceived and assessed by migrant women. In their study, Garbett et al.^25^ identified a ‘paradox of choice’ in decision-making, arguing that decisions that appear to be necessary for individuals to continue their journey, often compromise their health, well-being, autonomy, and dignity.

As many Venezuelan women migrate to neighbouring countries, often with young children in tow, motherhood becomes a central theme in their migration narratives.^26^ Since the seminal work on “transnational motherhood” by Hondagneu-Sotelo and Avila (1997) which highlighted the growing trend of mothers migrating internationally for labour purposes and the emotional and caregiving challenges of leaving their children behind, scholars have explored how migrant workers use various strategies to maintain connections with their families despite physical separation. One key strategy is combining paid work abroad with caregiving responsibilities back home, primarily through the regular sending of remittances.^27^, ^28^, ^29^, ^30^ Our study contributes to this literature by focusing on migrant women who flee to Brazil not in spite of being pregnant or having small children, but because of these circumstances. In cases of forced migration, like those included in our study, transnational motherhood is driven by practical necessities such as hunger, healthcare needs, poverty, or violence. This experience also profoundly impacts the health and well-being of migrant women.

Results from our survey show that almost one in three women left at least one child in Venezuela when migrating to Brazil. If we include in these numbers other family members, like parents, almost every migrant left family members behind. These women often become responsible for providing financial assistance to those who remain in their country of origin, as well as those under their care in the new place of abode, often despite receiving a very low income.^31^

Another important component of our analysis refers to the experiences of migrant mothers and their children in navigating and negotiating motherhood across different cultural, social, and geographical borders, as discussed previously by Oliveira (2018). Issues of identity, belonging, family dynamics, and socio-economic circumstances, as well as emotions such as worry, guilt and missing their children, shape not only parenting practices but also their emotional distress and perceptions of wellbeing.^32^^,^^33^ This resonates with a study conducted in Australia using the SF-36 questionnaire, which evaluates eight health dimensions and associates them with self-rated health, concluding that the most valued dimension for an individual when evaluating their health status is vitality, which involves energy and fatigue levels, followed by mental health (feelings of nervousness and depression), social functioning (willingness to participate in social activities), and emotional performance (ability to perform daily activities without interference from emotional problems).^34^ Interestingly, physical health dimensions were not statistically associated with the outcome assessed.^34^

Building on this debate, we examined how Venezuelan migrant women rated their health status. Self-rated health is a common measure in health surveys, yet it remains one of the most challenging indicators to interpret. Its value, however, lies in its ability to encapsulate subjective experiences, encompassing both physical and psychological dimensions and their manifestations, while reflecting the intricate relationship between the individual and the social world.

However, the value of this health construct lies precisely in summarising subjective experiences lived with physical and psychological aspects and their manifestations, reflecting the relationship between the individual and the social world.^8^

Venezuelan women rated their health status as better in Brazil than in Venezuela. Half of those who had declared their health condition as poor in Venezuela rated their health status as good in Brazil. This improvement may be related to new expectations, hope for a better life, and the fulfilment of immediate needs such as food, shelter, and access to health services.^31^^,^^35^ Although many of them lacked financial resources, almost all had shelter and access to daily meals. In Boa Vista, the main city of settlement, meals were provided through the joint efforts of religious entities, local civil society, and Operation Acolhida.^28^ Access to social programmes and some job opportunities, allowed them to send financial support to family members in Venezuela, contributing to their sense of well-being as they address aspects of caring for others, a significant contributor to worries and other emotional tensions. In addition, as health-related issues were a major motivation for coming to Brazil, Venezuelan women used health services twice as much as Brazilian women, indicating improved access to healthcare and contributing to a better self-assessment of their health after arriving in Brazil.^31^

Bahamondes et al. (2019) reported on Venezuelan women’s access to public health services and their satisfaction with the care received, particularly obstetric care in Pacaraima and Boa Vista.^36^ The right to have a companion of their choice during labour and delivery (companion law) was highly appreciated by them and is not routine practice in Venezuela.^23^^,^^36^^,^^37^ Yet, qualitative studies conducted by the same group of scholars with migrant women, health professionals and managers identified significant barriers to accessing primary care, particularly during the first prenatal consultation and when seeking contraceptive methods. Cultural and linguistic barriers made communication between migrant women and health professionals difficult, hindering their understanding of the information given.^38^^,^^39^

Collectively, the evidence from the literature and our results are consistent with what has been identified as the “healthy immigrant effect”^40^ which refers to perceptions that migrants have in relation to the improvement of their health status compared to the population in their country of birth; and to some extent to the population in the host country depending on the area of settlement. However, a number of studies also show that migrants’ health (perceived) outcomes may diminish over time and potentially worsen compared to the native population’s after years of settlement.^41^, ^42^, ^43^ This is particularly relevant to assess migrants who leave poor conditions due to hunger, poverty and violence as we have covered in our analysis. While migrants generally report better health upon arrival, this trend can be influenced by various factors, particularly in less affluent settings where poverty and limited resources pose additional challenges. Studies found a positive association between poorer self-rated health and the presence of diagnosed diseases, aligning with previous research on Brazilian women, which showed that a higher number of illnesses correlated with poorer self-rated health.^44^ However, in the current study, no significant differences were observed in self-rated health based on educational level, paid work, type of residence, immigration status, parity, or current pregnancy. Focusing on the health perceptions of migrant women, particularly forced migrants, the results of our study show that Venezuelan women who left children behind in Venezuela report lower levels of self-rated health. This aligns with previous research, which found that migrant mothers often face emotional distress, depression, and anxiety after migrating.^33^^,^^45^ The results also show that violence suffered in the course of migration and settlement in Brazil has also been associated with a worse self-assessment of the health status of Venezuelan women, as shown in other studies.^46^ This pattern is not unique to Venezuelan migrants and a high prevalence of violence in the migratory path of migrant women has been reported for several contexts, and can be as high as 40% for irregular migrants trying to enter the United States.^47^

Borders have also been reported as sites of violence, including instances of family separation.^48^ The militarisation, stricter control measures, and arbitrary closures—such as the nearly year-long closure of the Brazil–Venezuela border during the COVID-19 pandemic in 2020—drove an increase in informal crossings, heightening the risk of abuse and violence against migrants, particularly women and girls. Even after the border reopened, informal crossings persisted, as many women feared deportation and struggled to obtain passports or other identification documents for their children in Venezuela. Venezuelan migrant women have reported experiencing gender-based violence, xenophobia, and physical and psychological aggression both in and outside of shelters.^49^

Although the reported rates of violence among Venezuelan women are below 13%, it is important to consider the likelihood of underreporting due to language and legal barriers, as well as fear of denouncing a family member, partner, authorities, or another migrant. Notably, this study found that the highest incidence of violence, particularly physical violence, occurred within shared residences in Brazil. Married women in this study reported worse self-rated health and a higher prevalence of violence compared to single women. Recent research indicates that intimate partner violence (IPV) is highly prevalent in Latin America, affecting nearly one-third of women, according to the WHO (2021).^50^ This pattern is also evident among migrant women,^46^^,^^51^ as reflected in our data, which underscores the significant impact of violence on the health of migrant women.

The worse self-rated health in migrant women who left children in Venezuela and suffered violence on the way and/or in Brazil reinforces the complexity of the concept of health, which was sensitive to the suffering of this group of women in this study. Having children, however, acted as a protective factor, reducing the likelihood of low self-rated health when compared to childless migrant women. For the migrant women who left their families and suffered the impacts of separation and violence, being able to count on their loved ones was a psychological and emotional comfort.

The strength of these findings is compounded by the size of the sample and the representativeness of the survey, considering this is a hard-to-reach and follow population. To the best of the authors’ knowledge, there are no published studies on the impact of transnational motherhood on the self-perception of health of migrant women in this south-south migratory corridor.

As limitations, we acknowledge that choosing the RDS methodology for participant recruitment may have resulted in an overrepresentation of women residing in shelters, potentially due to easier communication channels, as well as other selection biases known to this method. The data collection took place in 2021 during the COVID-19 emergency, which limited our access to the field. The focus specifically on recent migration from Venezuela to Brazil (2018–2021) did not encompass other nationalities. Previous studies indicate that proximity to the border, frequency of communication with children remaining in the country of origin, and remittance sending may mitigate the psychological impact and suffering of mothers,^52^^,^^53^ although we did not specifically explore their influence on self-reported health. Additionally, data on violence are likely underreported due to closed questions and the limitations of epidemiological surveys for capturing such information. We also highlight the absence of data on other clinical dimensions and women’s mental health, which would enable better control of these factors in multivariate analyses. Finally, our cross-sectional approach limits our conclusions to the association level and cannot imply causality or direction of effects.

### Conclusion

The study analysed gender-related aspects within migration, such as transnational motherhood and encounters with violence, and explored their impact on individuals’ subjective health evaluations. This is innovative not only in terms of its methodological and conceptual approach and contextualisation but also in providing new insights into the strong correlation between self-assessed health and experiences of migration, particularly among migrant women. Although Venezuelan women rated their health better in Brazil than in Venezuela, multivariate analysis underscored a significant association between poorer self-rated health with the experience of leaving children behind in Venezuela and enduring violence during migration and in places of abode. These findings highlight the profound impact of migration-related stresses on health outcomes and underscore the urgent need for comprehensive support, protective policies, and health interventions to address the complex challenges faced by Venezuelan migrant women in Brazil.

## Contributors

Maria do Carmo Leal: Conceptualisation, methodology, writing original draft, review, and editing.

Thaiza Dutra Gomes de Carvalho: Conceptualisation, methodology, formal analysis, data curation visualization, writing original draft, review and editing.

Yammê Ramos Portella Santos: Methodology, formal analysis Data curation, investigation.

Rita Suely Bacuri de Queiroz: Data curation, investigation.

Paula Andrea Morelli Fonseca: Investigation, data curation, review and editing.

Antonio Augusto Moura da Silva: Conceptualisation, methodology.

Celia Landmann Szwarcwald: Conceptualisation, methodology.

Pía Riggirozzi: Writing original draft, review and editing.

## Data sharing statement

Data can be made available via the corresponding author.

## Declaration of interests

We declare no competing interests.
